# The MIF Antagonist ISO-1 Attenuates Corticosteroid-Insensitive Inflammation and Airways Hyperresponsiveness in an Ozone-Induced Model of COPD

**DOI:** 10.1371/journal.pone.0146102

**Published:** 2016-01-11

**Authors:** Kirsty E. Russell, Kian Fan Chung, Colin J. Clarke, Andrew L. Durham, Patrick Mallia, Joseph Footitt, Sebastian L. Johnston, Peter J. Barnes, Simon R. Hall, Karen D. Simpson, Malcolm R. Starkey, Philip M. Hansbro, Ian M. Adcock, Coen H. Wiegman

**Affiliations:** 1 Airway Disease Section, National Heart & Lung Institute, NIHR Respiratory Biomedical Research Unit at the Royal Brompton NHS Foundation Trust and Imperial College London, London, United Kingdom; 2 Airway Disease Infection Section, National Heart & Lung Institute, Imperial College London, London, United Kingdom; 3 RRI DPU, GlaxoSmithKline, Stevenage, United Kingdom; 4 Priority Research Centre for Respiratory Diseases, Hunter Medical Research Institute and The University of Newcastle, Newcastle, Australia; French National Centre for Scientific Research, FRANCE

## Abstract

**Introduction:**

Macrophage migration inhibitory factor (MIF) is an inflammatory cytokine associated with acute and chronic inflammatory disorders and corticosteroid insensitivity. Its expression in the airways of patients with chronic obstructive pulmonary disease (COPD), a relatively steroid insensitive inflammatory disease is unclear, however.

**Methods:**

Sputum, bronchoalveolar lavage (BAL) macrophages and serum were obtained from non-smokers, smokers and COPD patients. To mimic oxidative stress-induced COPD, mice were exposed to ozone for six-weeks and treated with ISO-1, a MIF inhibitor, and/or dexamethasone before each exposure. BAL fluid and lung tissue were collected after the final exposure. Airway hyperresponsiveness (AHR) and lung function were measured using whole body plethysmography. HIF-1α binding to the *Mif* promoter was determined by Chromatin Immunoprecipitation assays.

**Results:**

MIF levels in sputum and BAL macrophages from COPD patients were higher than those from non-smokers, with healthy smokers having intermediate levels. MIF expression correlated with that of HIF-1α in all patients groups and in ozone-exposed mice. BAL cell counts, cytokine mRNA and protein expression in lungs and BAL, including MIF, were elevated in ozone-exposed mice and had increased AHR. Dexamethasone had no effect on these parameters in the mouse but ISO-1 attenuated cell recruitment, cytokine release and AHR.

**Conclusion:**

MIF and HIF-1α levels are elevated in COPD BAL macrophages and inhibition of MIF function blocks corticosteroid-insensitive lung inflammation and AHR. Inhibition of MIF may provide a novel anti-inflammatory approach in COPD.

## Introduction

Macrophage migration inhibitory factor (MIF) is an inflammatory cytokine originally described as a T-cell mediated factor that suppressed the migration of macrophages and subsequently as a factor regulating macrophage host-defence functions [1, 2]. Increased expression and secretion of MIF has been reported in several acute and chronic inflammatory diseases such as sepsis [3], arthritis [4], asthma [5, 6] and lung cancer patients with COPD [7]. MIF is produced by a variety of inflammatory and immune cells and its expression is regulated by several different stimuli; however, its precise mechanism of action is still unclear [1, 2].

Chronic obstructive pulmonary disease (COPD) is characterised by airflow limitation and tissue destruction as exemplified by the presence of emphysema [8]. No murine model can recapitulate all the hallmark features of COPD but ozone-exposure and cigarette smoke-exposure can model aspects of COPD. Six-week ozone exposure of mice resulted in a COPD-like phenotype similar to that seen with more chronic 6 to 8 month cigarette smoke exposure. This was associated with emphysema-like enlargement of the alveolar spaces, chronic lung inflammation and enhanced levels of pro-inflammatory cytokines [9]. The inflammatory effects in the cigarette smoke-induced COPD model can vary with exposure time and COPD-like features, however the rapid intense 8–12 week model exhibits major characteristics of COPD including reduced lung function and emphysema-like lesions [10]. These models are also corticosteroid (CS)-insensitive, a main aspect of COPD and a critical issue with disease control [9, 10].

Under normoxic conditions, the continuous expression of the transcription factor, hypoxia inducible factor-1α (HIF-1α) is balanced by its degradation through the actions of prolyl-hydroxylases (PHD). However under hypoxic conditions, PHDs are inhibited and degradation reduced. This leads to HIF-1α stabilisation and subsequent nuclear translocation and transcription of target genes such as vascular endothelial growth factor (VEGF) [11, 12].

We hypothesised that MIF is involved in maintaining the chronic inflammatory process of COPD. We therefore investigated the role of MIF in the inflammation and pathophysiology of COPD by measuring MIF in patients with COPD and by studying the effect of a MIF inhibitor, (S,R)3-(4-hydroxyphenyl)-4,5-dihydro-5-isoxazole acetic acid methyl ester (ISO-1), in our chronic ozone-exposed mouse model of COPD. ISO-1 inhibits MIF tautomerase activity in a concentration-dependent manner with an IC_50_ of ~7μm [13], and has been previously shown to prevent airway hyperresponsiveness (AHR) in mouse ovalbumin (OVA)-challenge models [14].

Our study demonstrated enhanced MIF expression in the sputum and BAL macrophages of patients with COPD compared with control subjects. MIF expression correlated with that of HIF-1α in patients and in an animal model of COPD and in mouse lung HIF-1α binding to the *Mif* promoter was associated with enhanced MIF expression. ISO-1 attenuated ozone-induced cell recruitment, cytokine release and AHR in the mouse but did not affect measures of emphysema. These data suggest that MIF may drive COPD inflammation but not emphysema but clinical trials using anti-MIF approaches are needed to confirm this.

## Materials and Methods

### COPD Subjects

Aged matched groups of non-smokers (NS) and smokers (S) with normal lung function and COPD patients (GOLD stage II) were recruited. St Mary’s Hospital Local Ethics Committee approved the study (07\H0712\138). All subjects were aged 40–75 years; had no history of asthma or allergic rhinitis and were not atopic on skin testing; had no current or previous history of bronchiectasis, carcinoma of the bronchus or other significant respiratory disease (other than COPD); an absence of significant systemic disease; no COPD exacerbation or respiratory tract infection within the previous eight weeks; had a serum antibody titre to rhinovirus 16 <1.2 at screening and had not been treated with antibiotics, oral, inhaled or nasal topical steroids, long-acting β-agonists or tiotropium in the previous three months. COPD patients had an FEV_1_ 50–79% predicted normal value and β_2_-agonist reversibility <12%; an FEV_1_/FVC ratio <70% and were current or ex-smokers with at least 20 pack years cumulative smoking. Smokers had an FEV1 ≥80% predicted normal value, FEV1/FVC ratio >70% and were current or ex-smokers with at least 20 pack years cumulative smoking. In contrast, non-smokers had an FEV_1_ ≥80% predicted normal value, FEV_1_/FVC ratio >70%.

All subjects gave written informed consent and none were using inhaled or oral CSs. Subjects were recruited to an experimental rhinovirus (RV) infection study and the samples used were those collected at baseline prior to inoculation [15, 16]; all subjects were free of respiratory infection for 8 weeks prior to the study Sputum was induced [15] and cytokines measured by ELISA in sputum supernatant. Due to limited sample quantity not all patient samples were assessed (11 NS, 8 S and 12 COPD patients). Bronchoalveolar lavage (BAL) was performed by fibreoptic bronchoscopy; macrophages were isolated from BAL [16] and whole cell proteins were extracted and MIF was measured by ELISA (13 NS, 12 S and 12 COPD patients) (Table 1).

**Table 1:** Sputum and BAL participant details. FEV_1_: Forced expiratory volume in one second; FVC: forced vital capacity.

	*Non-smokers*	*Smokers*	*COPD*
*n*	13	12	12
*Gender (male/female)*	6/7	7/5	8/4
*Pack years*	0	32(21–51)**	39(25–57)***
*Age (years)*	59(46–71)	54(41–66)	59(44–72)
*FEV*_*1*_ *(%predicted)*	106±4	99±3*	65±2***
*FEV*_*1*_*/FVC (%) BAL cell count*	79±1 1.98±0.04	77±2 2.44±0.15	58±2***2.28±0.18*

* p<0.05

** p<0.01

*** p<0.001 compared to healthy control, data expressed as median and percentiles or mean±SEM.

In a separate cohort, aged-matched non-smokers and smokers with normal lung function and COPD subjects (GOLD stage I-III) were recruited. Cytokines in serum were measured by ELISA. The Royal Brompton and Harefield Hospital Trust Ethics Committee approved the study (09\H0801\85) and all subjects gave written informed consent. No subjects were using inhaled or oral CSs (Table 2).

**Table 2:** Serum participant details. FEV_1_: Forced expiratory volume in one second; FVC: forced vital capacity.

	*Non-smokers*	*Smokers*	*COPD*
*N*	14	22	25
*Gender (male/female)*	8/6	14/8	16/9
*Pack years*	0	28±3**	44±11***
*Age (years)*	51±2	59±2	70±2
*FEV*_*1*_ *(%predicted)*	105±4	86±3**	63±4***
*FEV*_*1*_*/FVC (%)*	98±3	84±3**	58±2***

* p<0.05

** p<0.01

*** p<0.001 compared to healthy control, data expressed as median and percentiles or mean±SEM.

### Mice models of cigarette and ozone exposure

The cigarette smoke model was performed at The University of Newcastle (Australia); the institution animal ethics committee approved all experiments. Female BALB/c mice were exposed to cigarette smoke for 75 minutes, twice a day, 5 times week for 6, 8 or 12 weeks using custom-designed, purpose-built nose-only, directed-flow inhalation and smoke exposure systems [10, 17]. Control groups were exposed to ambient air.

Ozone experiments were approved and performed under a British Home Office, UK Project License 70/7581 and approved by the Imperial College London institution animal ethics committee. Male C57BL/6 mice (Harlan, UK) were exposed to 3ppm of ozone generated from an ozoniser (model 500 Ozoniser, Sander, Germany) for 3 hours, twice a week for 6 weeks [18]. Control groups were exposed to ambient air.

### ISO-1 and dexamethasone treatments

ISO-1 (20mg/kg in 5% DMSO, Calbiochem, UK) and/or dexamethasone (2mg/kg, Sigma, UK) were given 1 hour (i.p.) before each ozone exposure. Air-exposed control mice received ISO-1 and/or dexamethasone treatment at the same time points.

### Pulmonary function analysis

Twenty-four hours after the final ozone exposure, mice were anesthetised with ketamine (100 mg/kg, Ketaset, Fort Dodge, USA) and xylazine (10 mg/kg, Xylacare, Animal Care, UK) i.p., were tracheostomised and placed in a plethysmograph (eSpira™ Forced Manoeuvers System, EMMS, UK) [18]. Functional residual capacity (FRC) was determined by Boyle’s law and lung compliance (C_chord_) was measured from the quasi-static pressure-volume manoeuvre. Total lung capacity (TLC) and the forced expiratory volume in first 75 milliseconds of exhalation (FEV_75_) were recorded during fast-flow volume manoeuvre.

### Measurement of airway hyperresponsiveness

Tracheostomised mice were ventilated (MiniVent, Hugo Sach Electronic, Germany) at 250 breaths/minute and tidal volume of 250μl. Transpulmonary pressure was assessed via an oesophageal catheter (EMMS, Hants, UK). Pulmonary airway resistance (R_L_) was recorded for 3-minutes after increasing concentrations (4-256mg/ml) of aerosolized acetylcholine (Sigma, UK). R_L_ was expressed as percentage change from PBS baseline (Sigma, UK). The acetylcholine concentration required to increase R_L_ by 100% from baseline was calculated (PC_100_), -log PC_100_ was taken as a measure of AHR.

### Bronchoalveolar lavage cells

After mice were sacrificed, BAL samples were obtained [9] and total cell counts calculated. Cytospin slides (Shandon Cytospin 4; Thermo Electron Corporation, USA) of BAL cells were stained using Diff-Quick kit (Reagena, Toivala, Finland) and differential cell counts performed in a blinded manner. In brief, following an overdose of aesthetic, mice were lavaged with one 0.8-ml aliquot of PBS via a 1-mm diameter endotracheal tube, and bronchoalveolar lavage (BAL) fluid was retrieved. Total cell counts and differential cell counts from slide preparations prepared by using a cytospin procedure and stained by Wright-Giemsa stain set (Sigma, UK) were determined under an optical microscope (Olympus BH2; Olympus Optical, Tokyo, Japan). At least 400 cells were counted per mouse and identified as macrophages, eosinophils, lymphocytes, and neutrophils according to standard morphology under ×400 magnification.

### RNA isolation

Total RNA was extracted from frozen lung tissue using an RNeasy mini kit (Qiagen, Crawley, UK) [19]. Transcript levels were determined by RT-qPCR (Corbett Research, Sydney, Australia) using SYBR^®^ Green PCR Master Mix Reagent (Qiagen, Crawley, UK), Primers are listed in Table 3.

**Table 3:** List of forward and reverse primers and PCR conditions.

*Gene*	*Primer*	*Sequence*	*PCR conditions*
*18S*	Forward Reverse	CTTAGAGGGACAAGTGGCG ACGCTGAGCCAGTCAGTGTA	20s@95°C, 30s@60°C, 30s@72°C x 30 cycles
*Kc*	Forward Reverse	CGCTGCTGCTGCTGGCCACCA GGCTATGACTTCGGTTTGGGTGCA	20s@95°C, 30s@60°C, 30s@72°C x 60 cycles
*Gmcsf*	Forward Reverse	TGGTCTACAGCCTCTCAGCA GCATGTCATCCAGGAGGTTC	20s@95°C, 30s@60°C, 30s@72°C x 60 cycles
*Ifng*	Forward Reverse	GTGGTTGACACTTAGTGGTCTC GGTGACATGAAAATCCTGCAGAGC	20s@95°C, 30s@60°C, 30s@72°C x 60 cycles
*Tnfa*	Forward Reverse	AGTTCTATGGCCCAGACCCT AGGGTCTGGGCCATAGAACT	20s@95°C, 30s@62°C, 30s@72°C x 60 cycles
*Mif*	Forward Reverse	CAAGCCCGCACAGTACATC AGGCCACACAGCAGCTTACT	20s@95°C, 30s@60°C, 30s@72°C x 60 cycles
*Mif* (Cig Smoke)	Forward Reverse	CGTGCACTGCGATGTACTGT CCATGCCTATGTTCATCGTG	20s@95°C, 30s@60°C, 30s@72°C x 60 cycles
*Hprt*	Forward Reverse	AGGCCAGACTTTGTTGGATTTGAA CAACTTGCGCTCATCTTAGGCTTT	20s@95°C, 30s@60°C, 30s@72°C x 60 cycles

### Protein isolation

Protein isolation was performed using a nuclear extraction kit (Active Motif Europe, Belgium) [20]. Concentrations were determined using a protein assay kit (Pierce Chemical, USA).

### Enzyme-linked immunosorbent assays

KC, GM-CSF, TNF-α, MIF and HIF-1α were quantified using commercially-available ELISA kits (R&D Systems Europe Ltd, UK) according to manufacturer’s instructions.

### Statistics

Data are expressed as mean±SD unless otherwise specified. The Kruskal-Wallis ANOVA test was used for comparisons of multiple groups with a post-test Mann-Whitney analysis performed if necessary. One-way ANOVA and Pearson correlation analysis were used for human sputum and BAL macrophage data. Grubbs test was used to determine outliers. A *p* value of <0.05 was accepted as significant.

## Results

### MIF protein expression is elevated in sputum from COPD patients

**Fig 1:**
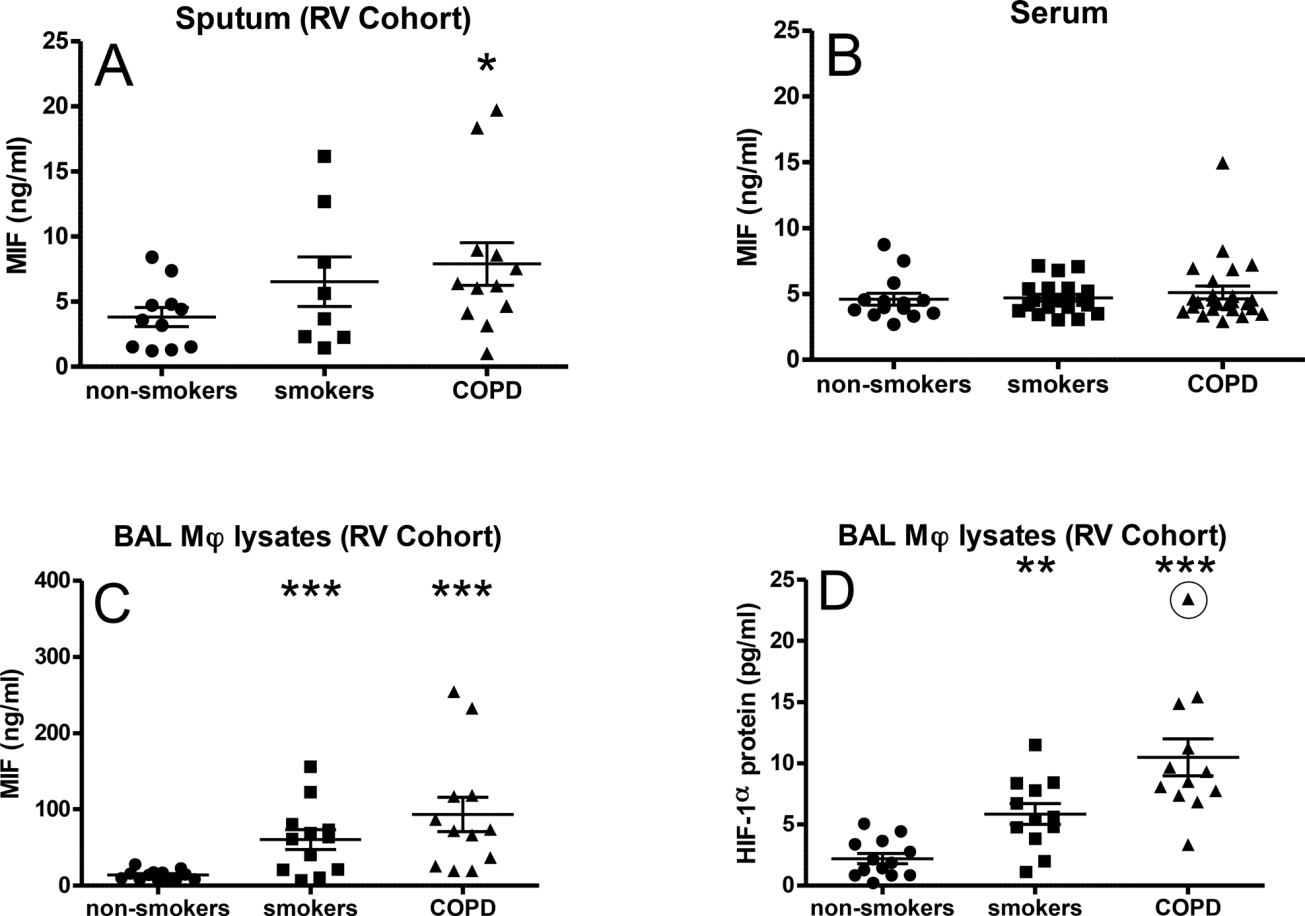
MIF and HIF-1α expression levels in COPD. MIF protein was measured in sputum (A; RV cohort), serum (B) and isolated BAL macrophages (C; RV cohort). HIF-1α protein concentration was measured in isolated BAL macrophages (D; RV Cohort). Data are expressed as mean±SEM. *p<0.05 and ***p<0.001 compared to non-smoking groups. Rhinovirus Infection (RV).

MIF protein levels were increased in sputum samples from COPD patients (7.9±2.4ng/ml, *p*<0.05, 95% CI[4.3, 11.5]) compared to non-smoking controls (3.8±1.6ng/ml, 95% CI[2.2, 5.4], Fig 1A). There was no significant difference in MIF expression between smokers (6.5±2.3ng/ml, 95% CI [2.0, 11.0]) and COPD patients.

There were no significant differences in serum MIF concentrations between groups (Fig 1B). Intracellular/cytoplasmic MIF concentrations were significantly elevated in BAL macrophages isolated from smokers (67.0±6.8ng/ml, *p*<0.001, 95% CI [21.5, 110.0]) and COPD patients (96.7±6.8ng/ml, *p*<0.001, 95% CI [63.3, 206.6]) compared to non-smoking controls (10.8±2.1ng/ml, *p*<0.001, 95% CI [7.6, 15.3], Fig 1C). There were no outliers as determined by Grubbs test.

### Correlation between MIF and HIF-1α protein expression in human BAL macrophages

**Fig 2:**
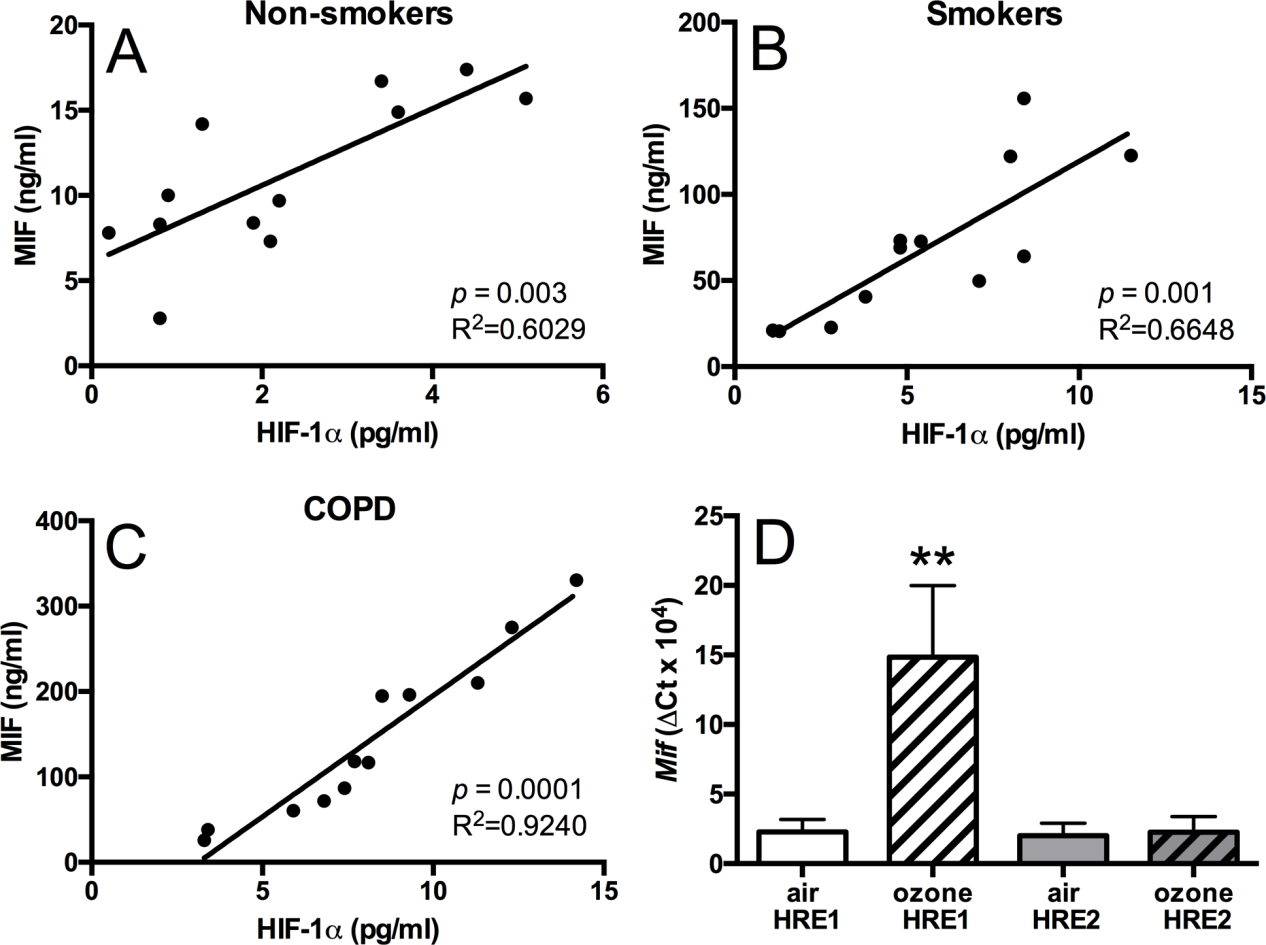
MIF and HIf-1α correlations in COPD and HIF-1 α regulation of *Mif* expression. Correlation analysis between HIF-1α and MIF protein concentrations in isolated BAL macrophages from non-smokers (A), smokers (B), and COPD patients (C). Chromatin immunoprecipitation analysis of HIF-1α binding to HRE1 and HRE2 sites in the *Mif* promoter in mouse lung tissue (D). Data are expressed as mean±SD for 6 animals per group. **p<0.01 compared to air controls.

HIF-1α protein expression was also elevated in BAL macrophages isolated from smokers (6.1±2.7pg/ml, *p*<0.001, 95% CI [4.3, 7.8]) and COPD patients (8.2±3.4pg/ml, *p*<0.001, 95% CI[6.5, 10.0]) compared to non-smokers (2.3±1.5ng/ml, 95% CI[1.5, 3.0] Fig 1D). HIF-1α and MIF levels were strongly correlated in all groups with the strongest correlation seen in the COPD group (Fig 2A–2C).

To test whether there was a mechanistic link driving these correlation we tested whether HIF-1α protein bound to HIF-1α response elements (HREs) in the MIF promoter. Due to the limited amounts of sample available from human BAL macrophages these experiments were conducted in ozone-treated lung samples where there was also a good correlation between HIF-1α and MIF expression. Ozone exposure induced binding of HIF-1α protein to the HIF-1α response element (HRE)1 site (-702bp) in ozone-exposed animals 15-fold (*p*<0.05), but not on the HRE2 (-1483bp) site (Fig 2D). The binding to the HRE1 site in ozone-exposed mice was associated with elevated *Mif* mRNA expression in the lung.

### Cigarette smoke-induced COPD model did not correlate with human results

To investigate the potential function of MIF in COPD, we used the two different *in vivo* mouse models of COPD for *Mif* gene expression and determined whether levels of MIF expression in these models were similar to those seen in human COPD. After 6 and 8 weeks of cigarette smoke exposure, *Mif* expression in lung tissue was reduced compared to air control by 51% and 36% respectively (Table 4). At 12 weeks, there was no longer a significant difference in *Mif* expression between the smoking and air control groups.

**Table 4:** Relative *Mif* mRNA expression in lungs of mouse models of COPD. Äct values of *Mif* expression in lung tissue relative to *Hprt* (cigarette model) and *18s* (ozone model) expression as measured by RT-qPCR. ND: not done.

	*Mif* mRNA expression		
	Week 6	Week 8	Week 12
Cigarette smoke	0.6±0.1	0.8±0.2	1.0±0.1
Air control	1.2±0.4	1.7±0.4	1.1±0.3
Ozone	0.5±0.1	ND	ND
Air control	0.2±0.1	ND	ND

In contrast, the 6-week ozone exposure model of COPD showed elevated levels of *Mif* expression in lung tissue (0.5±0.1 vs 0.2±0.1, *p*<0.05, Table 4) correlating with the human data reported here. Hence, we opted to use the ozone-exposure model for examining the role of MIF in COPD. There are some strain and gender differences in the *Mif* mRNA levels reported in the two animal models at baseline, however, in the context of this study the relative changes seen in the models and the relationship to the clinical samples are the most important.

### ISO-1 but not dexamethasone attenuated ozone-induced BAL cell numbers and cytokine release

**Fig 3:**
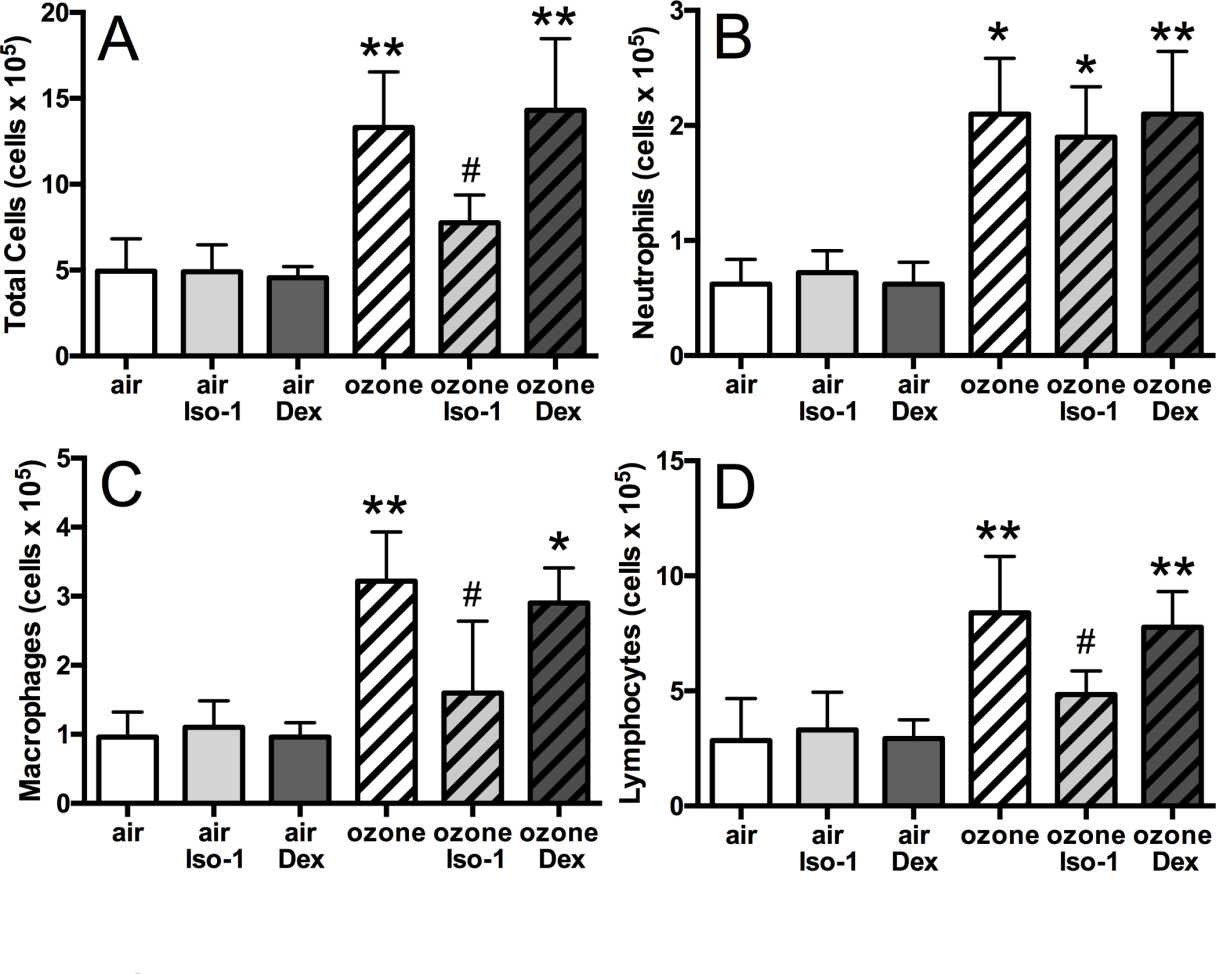
Effect of ISO-1 and dexamethasone on ozone-induced airway inflammatory cells. Total cell count (A), neutrophil (B), macrophage (C), and lymphocyte (D) counts in mouse BAL samples. Data are expressed as mean±SD for 6 animals per group. *p<0.05 and **p<0.01 compared to air controls, # *p*<0.05 compared to ozone exposed group.

In mice, ozone exposure led to an increase in total BAL cells (*p*<0.01, Fig 3A), reflected in an increase in neutrophils (3.4-fold, *p*<0.05), macrophages (3.4-fold, *p*<0.01) and lymphocytes (2.9-fold, *p*<0.01) compared to air-exposed controls (Fig 3B–3D). Dexamethasone had no effect on the number of BAL inflammatory cells in ozone-treated mice. In contrast, ISO-1 treatment attenuated the number of total BAL cells (1.7-fold decrease, *p*<0.05), with a reduction in macrophages (*p*<0.05) and lymphocytes (*p*<0.05) but not of neutrophils.

**Fig 4:**
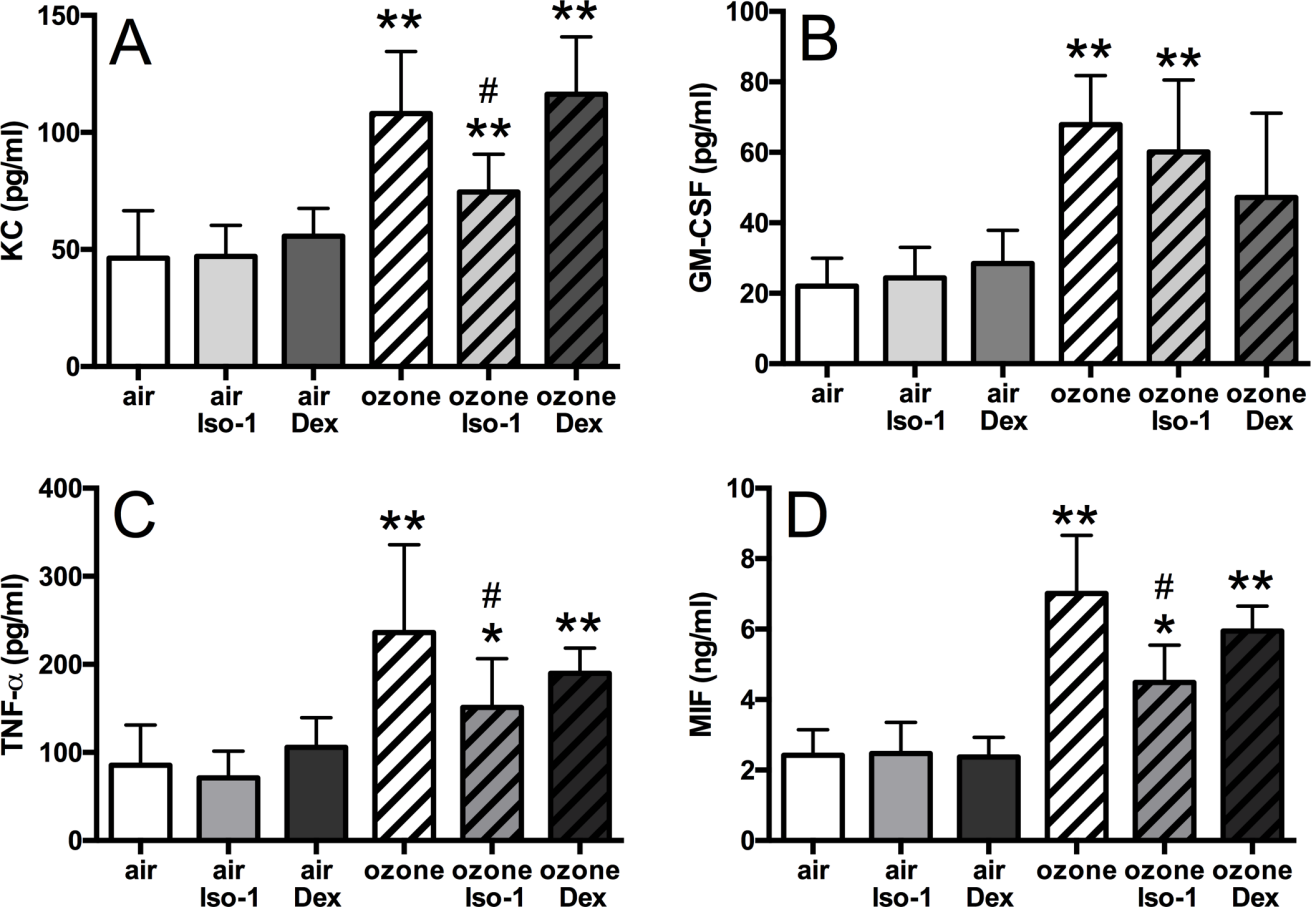
Effect of ISO-1 and dexamethasone on ozone-induced BAL inflammation. Cytokine protein levels in mouse BAL of ozone exposed and ISO-1- or dexamethasone-treated mice measured by ELISA. KC (A), GM-CSF (B), TNF-α (C) and MIF (D). Data are expressed as mean±SD for 6 animals per group. *p<0.05 and **p<0.01 compared to air controls, # *p*<0.05 compared to ozone exposed group.

BAL KC (2.3-fold), GM-CSF (3.1-fold), TNF-α (2.3-fold), and MIF (2.8-fold) were increased in ozone-exposed mice (*p*<0.01, Fig 4A–4D). BAL KC and TNF-α levels were reduced 1.4-fold and 1.6-fold respectively by ISO-1 treatment when compared to ozone alone, but levels did not return to baseline values compared to the air and ISO-1 treated control groups (*p*<0.05, Fig 4A and 4C). ISO-1 had no effect on BAL GM-CSF levels (Fig 4B). Ozone-induced BAL MIF concentration was reduced by 1.5-fold with ISO-1 pre-treatment (*p*<0.05, Fig 4D). In contrast, dexamethasone pre-treatment had no effect on ozone-induced increase of BAL KC, TNF-á or MIF levels (Fig 4A, 4C and 4D).

### Ozone-induced cytokine expression in the lung is insensitive to dexamethasone but sensitive to ISO-1 treatment

**Fig 5:**
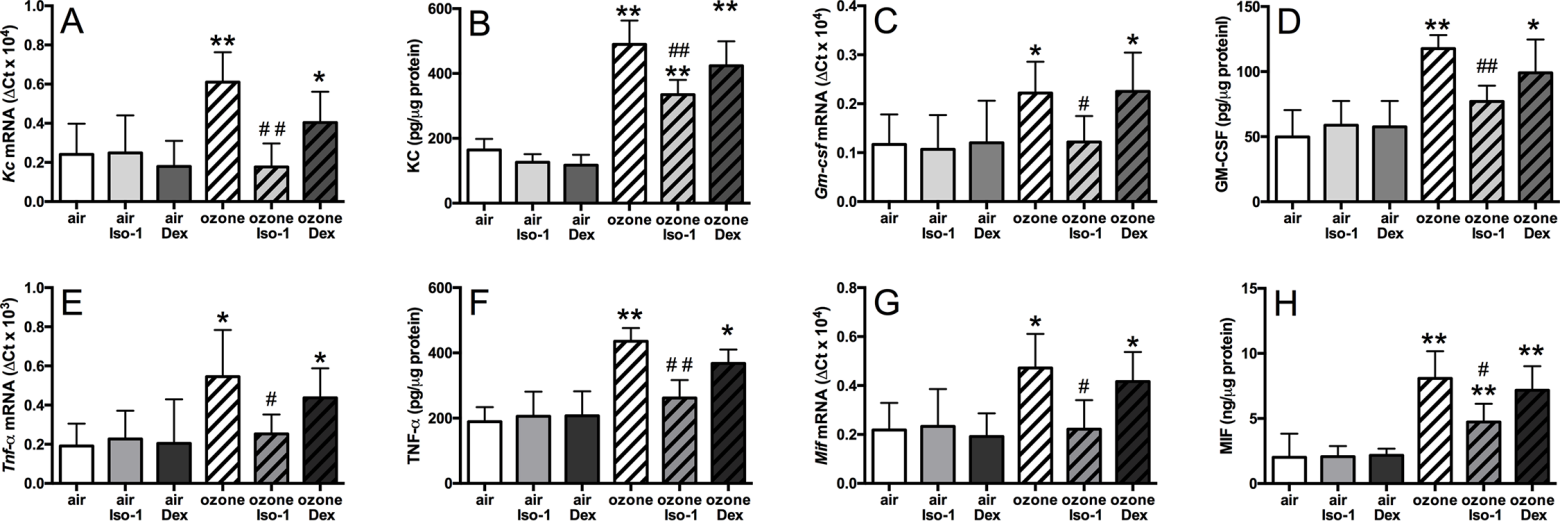
Effect of ISO-1 and dexamethasone on ozone-induced lung inflammation. Cytokine mRNA (A, C, E & G) and protein (B, D, F & H) expression levels in the lung of ozone exposed and ISO-1- or dexamethasone-treated mice. KC (A&B), GM-CSF (C&D), TNF-α (E&F), and MIF (G&H). Data are expressed as mean±SD for 6 animals per group. *p<0.05 and **p<0.01 compared to air controls, ^#^*p*<0.05 compared to ozone exposed group.

The mRNA levels, normalised to the *18S* housekeeping gene, of *Kc* (2.5-fold, *p*<0.01), *Gmcsf* (1.9-fold, *p*<0.05), *Tnfα* (2.3-fold, *p*<0.05) and *Mif* (2.2-fold, *p*<0.05) were elevated after ozone exposure (Fig 5A, 5C, 5E and 5G). Dexamethasone treatment did not affect the ozone-induced increase in gene expression levels. In contrast, ISO-1 treatment reduced the mRNA levels of each ozone-induced cytokine to basal levels (Fig 5A, 5C, 5E and 5G). Similarly, ozone exposure enhanced the protein levels of these cytokines in the mouse lung and these were suppressed by ISO-1 but not dexamethasone treatment (Fig 5B, 5D, 5F and 5H).

### ISO-1 attenuates ozone affected lung function and AHR

**Fig 6:**
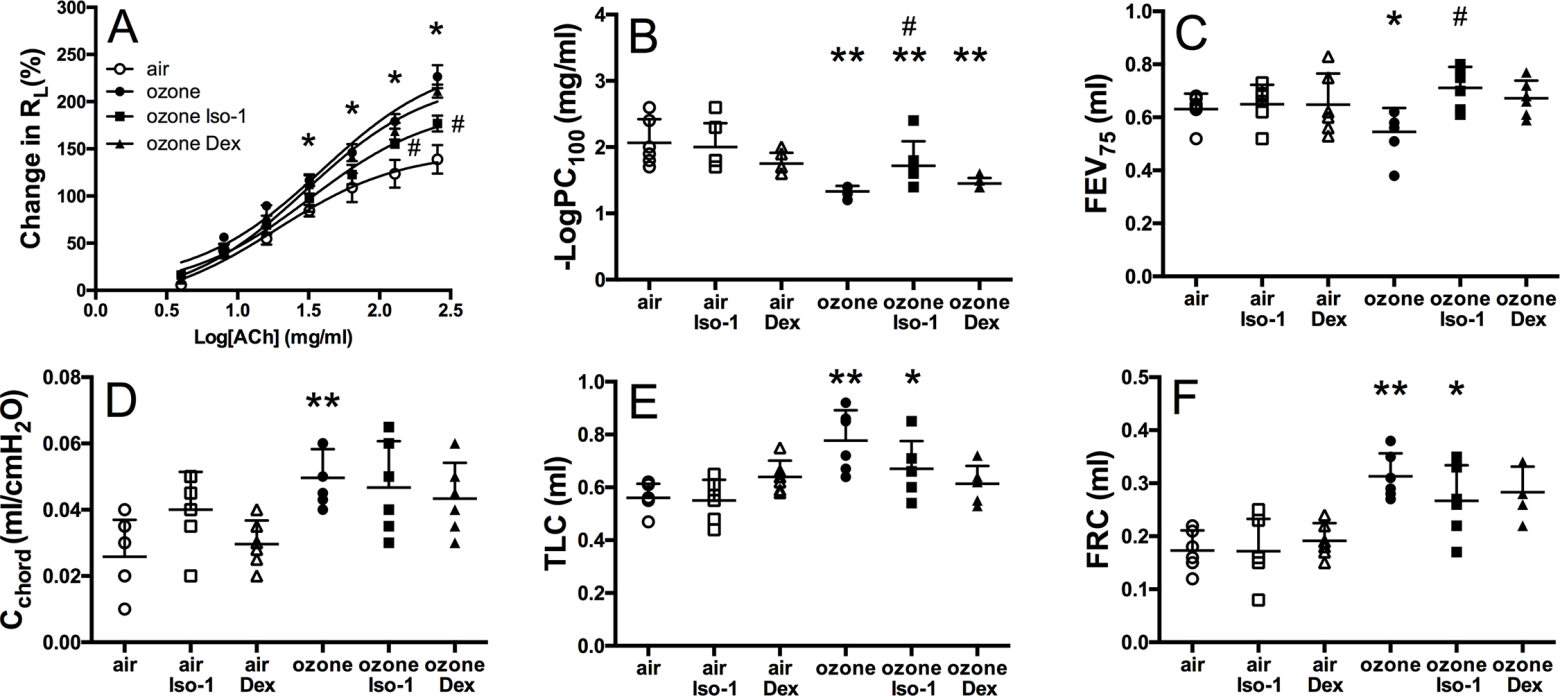
Effect of ISO-1 and dexamethasone on ozone-induced changes in AHR and lung function. Mouse lung function measurements of pulmonary resistance (R_L_; A), -logPC_100_ (B), FEV_75_ (C), lung compliance (C_chord_; D), total lung capacity (TLC; E) and functional residual capacity (FRC; F). Data are expressed as mean±SD for 6 animals per group. *p<0.05 and **p<0.01 compared to air controls, ^#^*p*<0.05 compared to ozone-exposed group.

Ozone alone increased R_L_, at the greatest concentration of acetylcholine, compared to air control (226.5±29.8% versus 139.0±37.3%, *p*<0.05) and was significantly reversed by ISO-1 (170.3±20.7%, *p*<0.05 Fig 6A) but not by dexamethasone (206.9±40.3%, *p*<0.05, Fig 6A). -LogPC_100_ is the concentration of acetylcholine needed to increase the pulmonary resistance by 100% from baseline. -LogPC_100_ was decreased 1.6-fold in ozone-exposed mice (*p*<0.01, Fig 6B). ISO-1 treatment reduced ozone-induced -LogPC_100_ 1.7±0.4mg/ml versus 1.3±0.1mg/ml, *p*<0.05) but this was not significantly affected by dexamethasone (1.5±0.1mg/ml; Fig 6B). In addition, FEV_75_ was decreased 1.2-fold in ozone-exposed mice compared to air controls (0.63±0.06ml versus 0.55±0.09ml, *p*<0.01, Fig 6C). This was significantly improved by ISO-1 but not by dexamethasone treatment (Fig 6C).

C_chord_ (Fig 6D), TLC and FRC (Fig 6E and 6F) were also significantly increased following ozone exposure (*p*<0.01). However, neither ISO-1 nor dexamethasone treatment had any significant effect on these parameters.

### Combination of ISO-1 and dexamethasone has no effect on ozone-induced inflammation

**Fig 7:**
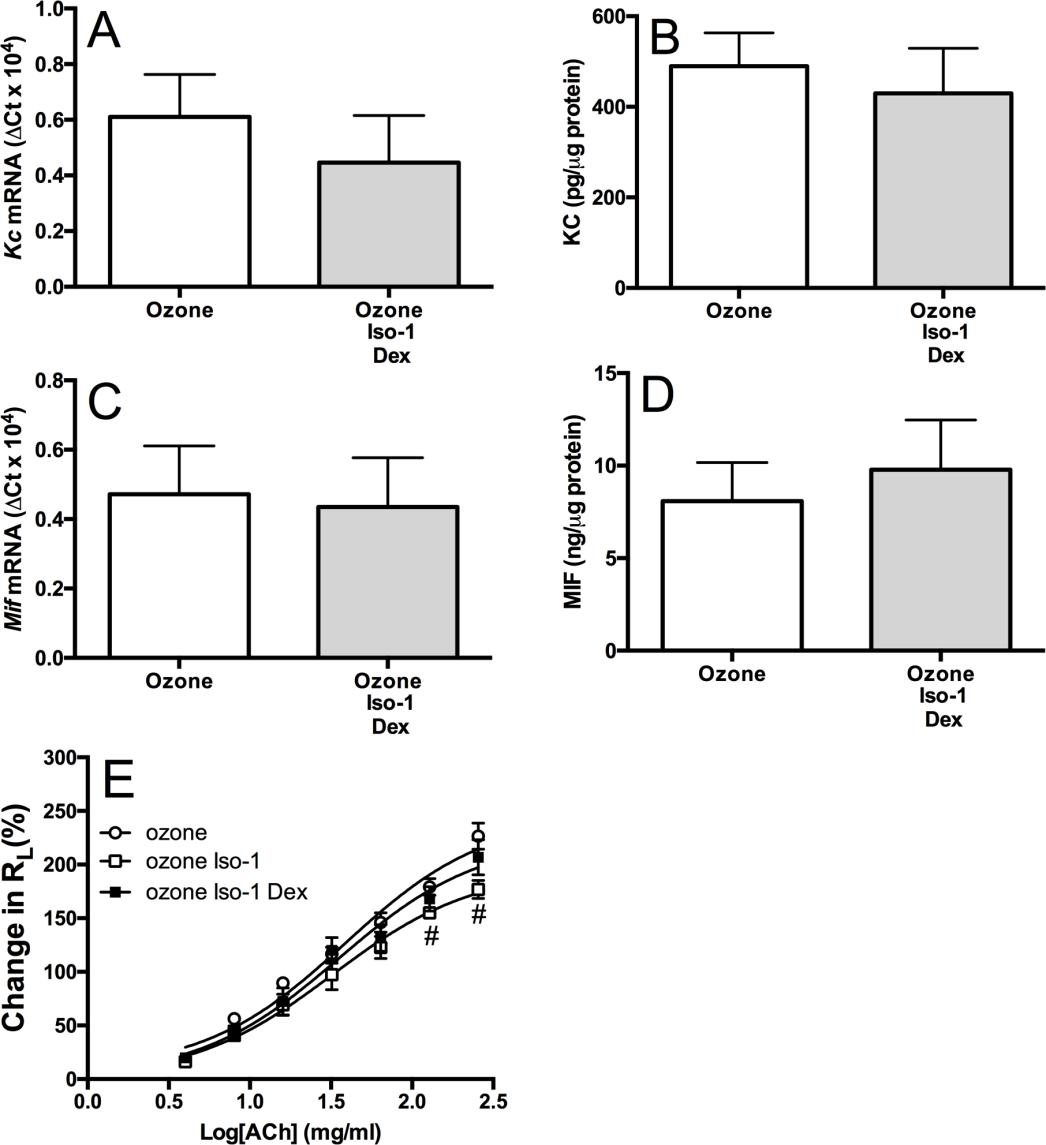
Effect of ISO-1 and dexamethasone in combination on ozone-induced lung inflammation. Cytokine mRNA (A & C) and protein (B & D) expression levels in the lung of ozone exposed and the combination of ISO-1- plus dexamethasone-treated mice. KC (A&B) and MIF (C&D). Pulmonary resistance (R_L;_ E) was also measured. Data are expressed as mean±SD for 6 animals per group. *p<0.05 and **p<0.01 compared to air controls, ^#^*p*<0.05 compared to ozone exposed group.

No additive anti-inflammatory effect on mediator release or BAL cell number was seen with ISO-1 and dexamethasone in combination compared to dexamethasone or ISO-1 alone. Also, the ozone-induced change in R_L_ was not affected by treatment with ISO-1 and dexamethasone in combination. However, the attenuation of R_L_ with ISO-1 alone was no longer evident in the presence of dexamethasone (Fig 7). These changes may reflect the numbers used per group as the studies were not powered to detect differences. Future studies should use more animals per group.

## Discussion

We demonstrate here that MIF protein expression is elevated in sputum samples, but not serum samples, from healthy smokers and COPD patients compared to healthy aged-matched non-smoking controls. In addition, we demonstrate that MIF expression is greater in BAL macrophages from COPD patients compared to control subjects. MIF expression correlates with HIF-1α which can enhance MIF expression by binding to specific regions within the MIF promoter. We examined two models of COPD for MIF expression, a 6 week ozone-exposure model and a cigarette smoke model (6, 8 and 12 weeks) to see which model replicated the human COPD results. MIF inhibition using ISO-1 prevented ozone-induced BAL, lung inflammation and AHR. In contrast, dexamethasone did not affect these parameters, confirming the corticosteroid insensitive COPD model. Overall, these data indicate that MIF may play a role in driving COPD inflammation and AHR but not emphysema.

In contrast to our results, MIF has been reported to be reduced in the blood of COPD patients compared to healthy and smoking subjects [21, 22]. These authors also report reduced MIF levels in the lungs of mice exposed to cigarette smoke for 6 months or more, which we also see in our more rapid cigarette smoke model, albeit at 12 weeks this reduction is no longer present. Moreover, these previous studies did not examine MIF levels in the lungs or airways of COPD patients or controls. Fallica and colleagues [21] report a decrease in serum MIF in COPD patients as a whole compared to healthy smokers; this is mainly due to a marked reduction in MIF levels in GOLD IV patients whilst the GOLD II and III patients have similar serum MIF levels as healthy smokers. The 25 COPD patients examined in our study were generally GOLD II patients with eight GOLD III subjects. Whether reduced MIF expression occurs in the more severe stages of COPD or in emphysematous patients will need to be further investigated.

In our ozone-induced model of COPD, we show increased MIF expression in the lungs at 6 weeks. Sauler and colleagues [22] showed increased lung MIF levels after 6-months of smoke exposure in mice but levels were markedly reduced at later time points. Conversely, Fallica et al., [21] demonstrate reduced MIF expression after 6 months of cigarette smoke exposure. Further comparisons between the smoking models and the ozone model are warranted particularly since the degree of inflammation and emphysema in both models is similar. Furthermore, how the data from these cigarette smoke models relate to the clinical results indicating increased MIF expression in sputum data also requires further experimentation. For this investigation, the ozone model reflected the higher expression of MIF in COPD patients; therefore we selected this model to study the inflammation role of MIF.

MIF and its receptors may also have a wider role in airways disease. Alveolar macrophage polarisation is modulated by smoking and this has important implications for COPD pathogenesis [23]. The fact that MIF expression is altered in COPD and modulates macrophage numbers in a murine model suggest that MIF may be implicated in smoking-induced macrophage reprogramming although this needs to be formally studied. In addition, MIF and its receptor CD74 have been shown to be increased in pulmonary arterial hypertension (PAH), a known COPD co-morbidity. Furthermore ISO-1 and anti-CD74 neutralizing antibodies partially reverse the development of PAH and inflammation in rats [24].

An anti-inflammatory effect of MIF inhibition has been previously reported in several rodent models of disease. Anti-MIF antibodies have been shown to be protective against endotoxemia [25], arthritis [26] sepsis [27] and OVA-induced allergic asthma [28]. In addition, *Mif* knockout mice also showed less inflammation in models of atopic dermatitis [29] and endotoxemia [30]. To our knowledge, this is the first time that MIF antagonism has been shown to have an anti-inflammatory effect in a corticosteroid-resistant mouse model of COPD and this also provides evidence that targeting MIF may be a useful therapeutic approach for patients with this disease. Chen *et al*. also reported on the effect of ISO-1 in the corticosteroid-sensitive mouse OVA-challenged model of airway inflammation [14].

COPD is characterised as neutrophilic and low doses of LPS in mice induces a neutrophil-rich lung inflammation [31], which is prevented by anti-MIF antibodies [32]. ISO-1 significantly suppressed ozone-induced total cellular recruitment, mostly as a result of reductions in BAL lymphocyte and macrophage but not neutrophil numbers. Magalhaes and colleagues also reported a reduction in AHR (Penh) in MIF knockout mice in response to metacholine in an OVA model of asthma [33]. Our data further supports the anti-inflammatory role of MIF inhibition, as we report a reduction in AHR (pulmonary resistance) however using a different model and AHR measurement. In contrast, others did not show effect of ISO-1 on inflammation in either OVA-challenged or LPS-treated mouse models [34].

Corticosteroid insensitivity is a major aspect of COPD and severe asthma [35]. The unique function of MIF in counter-regulating the function of corticosteroids [25] has driven research in many corticosteroid insensitive inflammatory diseases in an attempt to restore steroid-sensitivity, improve disease control, reduce exacerbations, disease progression and lower the doses of oral corticosteroid prescribed. We therefore examined whether combined ISO-1 and dexamethasone treatment would result in an enhanced anti-inflammatory effect in the lung. Although Chen *et al*. found that ISO-1 and dexamethasone separately had a comparable anti-inflammatory effect in the OVA-induced AHR model, they did not examine the effect of combined treatments [14]. In contrast, we found that the effects of ISO-1 and dexamethasone were not comparable and that ISO-1 pre-treatment had a more potent anti-inflammatory effect than dexamethasone in the ozone-induced model reflecting the corticosteroid-insensitive nature of this model. ISO-1 and dexamethasone treatment in combination showed no enhanced or additive anti-inflammatory effects on any of the ozone-induced parameters measured, including AHR or cytokines released in BAL fluid, indicating that MIF is unlikely to activate corticosteroid-associated pathways in this model.

We have previously demonstrated that chronic ozone exposure resulted in the activation of hypoxia-induced inflammatory pathways [20]. We now show that HIF-1α can bind to the native *Mif* promoter at specific HREs and thereby provide a mechanism by which *Mif* expression is enhanced in the lungs of ozone-exposed animals. Under hypoxic conditions, MIF inhibited dexamethasone-suppressed HIF-1α expression and also enhanced HIF-1α target gene expression in a positive feedback loop [36]. In support of a positive feedback loop for HIF-1α and MIF, we found that the increased levels of both proteins correlated in BAL macrophages from healthy (non-smokers), smokers and COPD patients. Furthermore in support of an article published by Gaber *et al*., we also observed a suppression of HIF-1α protein levels when MIF was inhibited by ISO-1 [36].

A previous study Baugh JA et al. [37] demonstrated that HIF-1α, acting through an HRE at +25 in the 5'UTR of the MIF gene, is a potent inducer of MIF expression. They also reported that this effect is amplified by hypoxia-induced degradation of cAMP responsive element binding protein (CREB). CREB expression is enhanced in COPD patients and a poor response to corticosteroid therapy may be related to increase CREB-associated signalling [38]. How MIF interacts with this pathway to modulate corticosteroid signalling in COPD patients requires further investigation. The interaction of MIF with the NF-κB pathway and the modulation of neutrophil apoptosis also requires further studies. NF-κB is activated in COPD patients [39] and this has been associated with a reduction in sputum neutrophils undergoing spontaneous apoptosis in COPD patients [40].

There are several limitations of the current study. We were unable to demonstrate a significant effect of ISO-1 on indicators of emphysema in the mouse model although AHR and inflammation were affected. This highlights the importance of COPD phenotyping in understanding the disease process. It will be important to perform clinical studies with MIF-targeting agents in order to fully define a role for MIF in COPD inflammation. Furthermore, the results to date do not exclude a role for the D-dopachrome tautomerase (D-DT or MIF-2) in driving COPD inflammation. Finally, we only used female mice in this study and further studies using male mice are warranted as males make up more than 50% of COPD patients due to previous smoking habits.

In conclusion, in our studies investigating the role of MIF in COPD inflammation and in inflammation and AHR in a mouse model of COPD we demonstrate a link between heightened MIF expression and COPD inflammation. In the ozone-induced corticosteroid-insensitive murine model of COPD, lung inflammation was attenuated by treatment with the MIF inhibitor, ISO-1. This supports a pro-inflammatory role for MIF in driving steroid-insensitive lung inflammation and cellular recruitment and infiltration to the lungs. However, ISO-1 treatment had no effect on suppressing ozone-induced neutrophilia and did not reverse corticosteroid insensitivity or emphysema, suggesting that MIF is not the primary driver of neutrophilia, steroid insensitivity or emphysema in this COPD model. However, clinical trials in specific subsets of COPD patients will need to be conducted using anti-MIF agents will be required to confirm this data.
